# Expression of miRNAs in Papillary Thyroid Carcinomas Is Associated with *BRAF* Mutation and Clinicopathological Features in Chinese Patients

**DOI:** 10.1155/2013/128735

**Published:** 2013-04-11

**Authors:** Yun Sun, Shuang Yu, Yuanyuan Liu, Fen Wang, Yujie Liu, Haipeng Xiao

**Affiliations:** ^1^Department of Endocrinology, First Affiliated Hospital, Sun Yat-sen University, 58 Zhongshan Road 2, Guangzhou 510080, China; ^2^Department of Pathology, First Affiliated Hospital, Sun Yat-sen University, 58 Zhongshan Road 2, Guangzhou 510080, China; ^3^Department of Breast Surgery, Sun Yat-sen Memorial Hospital, Sun Yat-sen University, 107 Yanjiang West Road, Guangzhou 510120, China

## Abstract

MicroRNAs (miRNAs) dysregulation has been shown to play a critical regulatory role in papillary thyroid carcinomas (PTCs). *BRAF* mutation is associated with poor clinicopathological outcomes in PTC. In order to identify a possible association between dysregulated miRNA expression and *BRAF* mutation as well as clinicopathological features in Chinese patients with PTC, we examined the expression levels of five reported dysregulated miRNAs (miRNA-221, miRNA-222, miRNA-146b, miRNA-181, and miRNA-21) and determined *BRAF* mutation status in 52 patients with PTC and 52 patients with benign thyroid nodules (BTNs). The expression levels of all five miRNAs were significantly increased in PTC when compared to BTN. The *BRAF* mutation occurred more frequently in PTC cases with advanced TNM stage. Importantly, miRNA-221, miRNA-222, miRNA-146b, and miRNA-181 expression levels were significantly higher in PTC patients with *BRAF* mutation. In addition, enhanced expression of miRNA-221 and miRNA-222 was found in patients with cervical lymph node metastasis and advanced TNM stage. Increased expression of miRNA-221 and miR-181 was evidenced in patients with larger tumors. These findings showed a potential role of this distinct profile of miRNAs in differentiating PTC from BTN. *BRAF* mutation might regulate or interact with miRNA in the pathogenesis and progression of PTC.

## 1. Introduction

Thyroid cancer is the most prevalent endocrine malignancy (accounting for >92% endocrine malignancy), which can be classified histologically into follicular epithelial cell-derived papillary thyroid cancer (PTC), follicular thyroid cancer (FTC), anaplastic thyroid cancer (ATC), and parafollicular C-cell-derived medullary thyroid cancer (MTC) [1, 2]. In the past several decades, the incidence of thyroid cancer worldwide has been steadily increasing, and this rising is mainly attributed to the increased diagnosis of PTC [2–9]. PTC is the most common histological type of thyroid cancers and accounts for approximately 80% of reported cases [10]. The overall prognosis of PTC patients is excellent after appropriate treatment. However, the mortality of PTC with aggressive clinicopathological features was much higher when compared with PTC without these clinicopathological features [1]. Therefore, it is of great importance to identify biomarkers that are associated with aggressive clinicopathological features of PTC, and these biomarkers might also serve as potential pharmacological targets for PTC.

*BRAF* mutation is the most common genetic alteration in thyroid malignances, exclusively existing in PTC and PTC-derived ATC, but not in FTC and MTC. This mutation is found in about 45% of sporadic PTC. Numerous studies have consistently shown that *BRAF* mutation, which upregulates thyroid cell division and proliferation by activating MAPK pathway, is mutually exclusive with other common genetic alterations such as the rearrangement in transformation/papillary thyroid carcinoma (RET/PTC) signal and *Ras* mutation [11–13]. Importantly, many studies have proved that *BRAF* mutation is correlated with high-risk clinicopathological characteristics, such as larger tumor size, extrathyroidal invasion, local lymph node metastasis, distant metastasis, and advanced disease stages, suggesting that it is not only an independent oncogenic event for PTC tumorigenesis, but also involved in progression of PTC [14–16].

MicroRNAs (miRNAs) are small noncoding RNA molecules, about 21–25 nucleotides in length, which negatively regulate gene expression at the posttranscriptional level. More than 1000 miRNAs are found in humans and up to one-third of the total human mRNAs are predicted to be miRNA targets [17]. Aberrant miRNA expression has been described in a variety of tumors, including PTC [17–21]. He et al. reported that 17 miRNAs were upregulated in PTC compared to adjacent normal tissue and defined five miRNAs (miRNA-221, miRNA-222, miRNA-146b, miRNA-181, and miRNA-21) being able to predict cancer status [19]. Tetzlaff et al. revealed the upregulation of miRNA-21, miRNA-31, miRNA-221, and miRNA-222 by real-time RT-PCR in PTC compared to multinodular goiter [20]. Among the various studies reported, the expression of 5 miRNAs, including miRNA-221, miRNA-222, miRNA-146b, miRNA-181, and miRNA-21, has been shown to be dysregulated [22]. In our study, we sought to further validate the expression of these 5 previously reported miRNAs in differentiating PTC from benign thyroid nodules (BTNs) in Chinese patients. Moreover, the associations between miRNAs and *BRAF* mutation, as well as other clinicopathological features, will be addressed in the present study. 

## 2. Patients and Methods

### 2.1. Samples Selection and RNA/DNA Extraction

Data of patients (shown in Table 1), archival FFPE surgical samples previously diagnosed as PTC (52 samples) and BTN (52 samples), were obtained from the First Affiliated Hospital and Cancer Center of Sun Yat-sen University (Guangzhou, China). All samples of PTC characterized in this study were the classic variant. Samples of BTN included solitary nodule and multinodular goiter. Informed consents were provided by all subjects and the study protocol was approved by the ethics committee of Sun Yat-sen University. For RNA isolation, eight 10 *μ*m sections per sample were obtained from the selected block for extraction of miRNA with the miRNeasy FFPE kit (QIAGEN, Hilden, Germany). Genomic DNA was extracted from FFPE samples using the QIAamp DNA FFPE Tissue Kit (QIAGEN). The RNA and DNA yields were determined using the NanoDrop ND-1000 spectrophotometer (Nanodrop, USA).

### 2.2. qRT-PCR

A panel of 5 miRNAs (miRNA-221, miRNA-222, miRNA-146b, miRNA-181, and miRNA-21) was selected based on previous reports [17–22]. Quantification of these five miRNAs was performed by using miScript PCR System (QIAGEN) as previously described [23]. In brief, each 5 *μ*L sample of dissolved RNA was polyadenylated and reverse-transcribed to cDNA in a final volume of 20 *μ*L using the miScript Reverse Transcription kit (QIAGEN). Real-time PCR was performed in duplicate using the miScript SYBR Green PCR kit (QIAGEN) with the ABI prism 7500 sequence detection system (Applied Biosystems, Foster City, CA, USA). The miScript Primer Assays are part of the miScript PCR System for miRNA detection and quantification. The U6 snRNA was used as the reference for miRNA expression. Relative quantitation of miRNA expression was performed by the comparative CT method [24].

### 2.3. *BRAF* Mutation Detection

Mutations in the *BRAF* gene were identified by direct DNA sequencing. For sequencing, exon 15, which contains the locus of the T1799A mutation, was amplified using primer pairs as previously reported [23].

### 2.4. Statistical Analysis

All statistical analyses were performed using Statistical Package for the Social Sciences (SPSS) software (version 16.0, Chicago, IL, USA). The Mann-Whitney *U* test was used to determine the significance of different levels of miRNA expression. Receiver operating characteristic (ROC) curves were used to analyze the diagnostic utility of different markers. Chi square test was used to identify possible associations between *BRAF *mutation and clinicopathological features of PTC patients. All *P* values were two-sided and a *P* value less than 0.05 was considered statistically significant. 

## 3. Results

### 3.1. Expression Profiles of Five miRNAs in PTC versus BTN and Their Predictive Value

**Figure 1:**
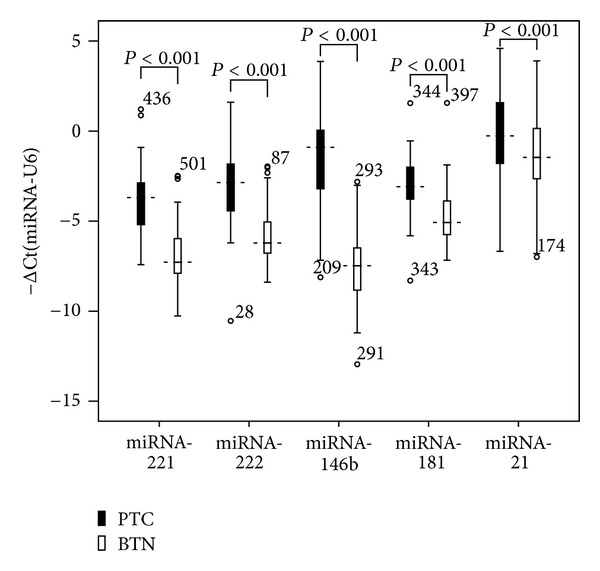
Box plots of miRNA expression levels of patients with PTC (*n* = 52) and BTN (*n* = 52). The levels of miRNA-221, miRNA-222, miRNA-146b, miRNA-181, and miRNA-21 were significantly higher in PTC patients than in BTN patients (*P* < 0.05 for all comparisons). All indicated *P* values were determined by the Mann-Whitney *U* test.

Expression of five miRNAs (miRNA-221, miRNA-222, miRNA-146b, miRNA-181, and miRNA-21) in FFPE tissues from patients with PTC or BTN was measured by qRT-PCR. All these five miRNAs were significantly higher in PTCs than those in BTNs (20.98 ± 46.52 versus 2.51 ± 4.66, 15.82 ± 26.07 versus 2.03 ± 3.24, 195.67 ± 426.14 versus 2.87 ± 5.74, 5.98 ± 11.20 versus 2.82 ± 11.16, and 14.78 ± 23.98 versus 3.44 ± 12.03, resp., all *P* values < 0.001) (Figure 1). 

**Figure 2:**
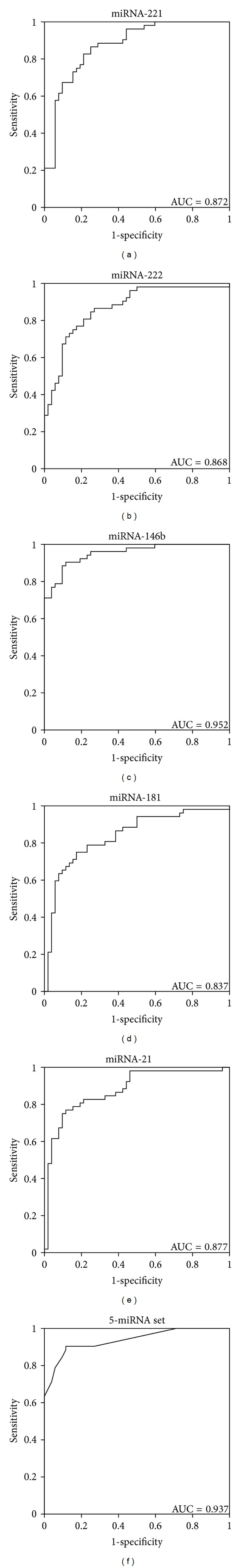
ROC curve analyses of miRNA-221 (a), miRNA-222 (b), miRNA-146b (c), miRNA-181 (d), miRNA-21 (e), and the 5-miRNA set (f) in the discrimination of patients with PTC from patients with BTN. The AUCs for miRNA-221, miRNA-222, miRNA-146b, miRNA-181, miRNA-21, and the 5-miRNA set were 0.872, 0.868, 0.952, 0.837, 0.877, and 0.937, respectively.

To evaluate the diagnostic value of these five miRNAs for PTC, ROC curve analysis was performed. Individually, the AUCs for miRNA-221, miRNA-222, miRNA-146b, miRNA-181, and miRNA-21 were 0.872 (cutoff value 0.022, sensitivity 82.7%, specificity 78.8%), 0.868 (0.069, 71.2%, 88.5%), 0.952 (0.021, 90.4%, 88.5%), 0.837 (0.079, 71.2%, 84.6%), and 0.877 (0.071, 76.9%, 88.5%), respectively (Figures 2(a)–2(e)). The 5-miRNA set had an area under the ROC curve (AUC) of 0.937 (95% confidence interval (CI) = 0.891–0.983), with a sensitivity of 90.4% and a specificity of 88.5% at the cutoff value of 0.471 (Figure 2(f)).

### 3.2. *BRAF* Mutation and Clinicopathological Features of PTC

When assessing the relationship between *BRAF* mutation and clinicopathological features, we found that *BRAF* mutation occurred more frequently in PTC patients (19/52) with advanced TNM stage (*P* = 0.014). However, other clinicopathological features, including tumor size and cervical lymph node metastasis, were not significantly different between patients with or without *BRAF* mutation. 

### 3.3. Differences in miRNA Expression in PTC Patients with and without *BRAF* Mutation

**Figure 3:**
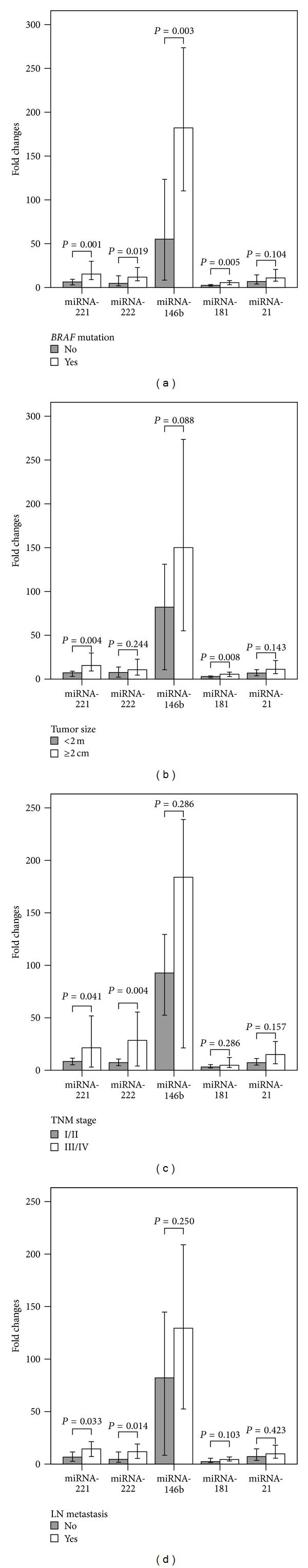
Differences in miRNA expression in PTC patients with different clinicopathological features. miRNA-221, miRNA-222, miRNA-146b, and miRNA-181 expression levels were significantly higher in *BRAF* mutation group than in non-*BRAF* mutation group (*P* < 0.05 for all comparisons). miRNA-21 expression level was not significantly different between the two groups (*P* = 0.104) (a). The levels of miRNA-221 and miRNA-181 were significantly higher in patients with tumor diameter ≥2 cm than <2 cm (*P* = 0.008 and *P* = 0.004, resp.) (b). The overexpression of miRNA-221 and miRNA-222 was found in patients with advanced TNM stage (*P* = 0.004 and *P* = 0.041, resp.) (c) and lymph node metastasis (*P* = 0.033 and *P* = 0.014, resp.) (d). Statistically significant differences were determined by the Mann-Whitney *U* test.

PTC patients were further divided into *BRAF* mutation group and non-*BRAF* mutation group and the expression of miRNA was compared. The miRNA-221, miRNA-222, miRNA-146b, and miRNA-181 expression levels were significantly higher in *BRAF* mutation group than in non-*BRAF *mutation group (miRNA-221, 15.29 (2.70–62.02) versus 6.36 (0.68–269.35), *P* = 0.001; miRNA-222, 11.82 (2.73–43.93) versus 4.73 (0.04–168.94), *P* = 0.019; miRNA-146b, 182.19 (10.70–1123.18) versus 55.11 (0.72–2917.96), *P* = 0.003; and miRNA-181, 5.58 (2.08–18.86) versus 2.46 (0.09–80.72), *P* = 0.005). However, there was no significant difference in miRNA-21 expression level between the two groups (*P* = 0.104) (Figure 3(a)). 

### 3.4. Differences in miRNA Expression in PTC Patients with Different Clinicopathological Features

We next accessed the differences in the expression levels of these 5 miRNAs in PTC patients with different clinicopathological characteristics (Table 2). The levels of miRNA-221 and miRNA-181 were significantly higher in patients with tumor diameter ≥2 cm than <2 cm (*P* = 0.004 and *P* = 0.008, resp.) (Figure 3(b)). The expression levels of miRNA-221 and miRNA-222 were significantly higher in PTCs with advanced tumor-node-metastasis (TNM) stage (*P* = 0.004 and *P* = 0.041, resp.) (Figure 3(c)). In addition, patients with lymph node metastasis had higher expression levels of miRNA-221 and miRNA-222 (*P* = 0.033 and *P* = 0.014, resp.) (Figure 3(d)). There were no significant differences in these miRNA levels in PTC patients with other clinicopathological parameters, including age, gender, tumor multicentricity, heteromorphic nucleus, thyroid globulin quantification, and AMES (risk definition including age, metastases, extent, and size) score (Table 2).

## 4. Discussion

In the present study, we have reported the association of 5 previously reported miRNAs and *BRAF* mutation and the clinicopathological features in Chinese patients with PTC. We showed that the expression levels of these five miRNAs (miRNA-221, miRNA-222, miRNA-146b, miRNA-181, and miRNA-21) were significantly higher in FFPE tissues from PTCs than BTNs. miRNA-221, miRNA-222, miRNA-146b, and miRNA-181 expression levels were significantly higher in PTC patients with *BRAF* mutation. Enhanced expression of miRNA-221 and miRNA-222 was found in patients with cervical lymph node metastasis and advanced TNM stage. Increased expression of miRNA-221 and miR-181 was evidenced in patients with larger tumors. 

Thyroid nodule is a common entity in the clinic. Although most of the thyroid nodules are benign, 5% to 30% of nodules are still malignant and require surgical intervention [25]. At present, pathological study is the golden criteria of diagnosis of thyroid nodules. However, there are several limitations in the pathological diagnosis of thyroid nodules, including inadequate sampling, indeterminate result, and possible misdiagnosis [26]. During the past decades, gene detection techniques have developed considerably, which make it possible to further explore tumorigenesis and progression of PTC at molecular level and to discover potential diagnostic and prognostic biomarkers. Among these techniques, detection of some specific miRNA expression levels is a promising method to provide information for molecular diagnosis of PTC [18–20]. In our study on utility of miRNA profiling, we have correlated this data with pathology of the surgical specimen, a gold standard of diagnosis, and we believe that our findings are certainly helpful in providing potentially diagnostic biomarker for PTC before operation. Our results demonstrated that each of these 5 miRNAs or the 5-miRNA set (miRNA-221, miRNA-222, miRNA-146b, miRNA-181, and miRNA-21) could be used as a potential biomarker to differentiate PTC from BTN, with highest diagnostic value for miRNA-146b (sensitivity of 90.4% and specificity of 88.5% at an AUC of 0.952) and 5-miRNA set (sensitivity of 90.4% and specificity of 88.5% at an AUC of 0.937). Consider that the upregulation level of miRNA-146b is not consistently reported [22]. Therefore, we suggest that use of the 5-miRNA set as a potential diagnostic biomarker for PTC might be more helpful. In order to further validate the potential diagnostic value of miRNA in the preoperative evaluation of thyroid nodules, we will analyze the proposed miRNAs data in “intermediate” or “suspicious” FNA cases based on current results in our future prospective study. 

*BRAF* mutation is the most common mutation in PTC, which leads to a constitutive activation of the MAPK pathway. Activation of this pathway is a common and important mechanism in genesis and progression of human cancers through upregulating cell division and proliferation [11–13]. A number of studies have reported that *BRAF* mutation was associated with aggressive PTC characteristics, such as extrathyroidal extension, lymph node metastasis, tumor recurrence, decreased survival, and need for cervical reoperation [14–16]. In our present study, we found that *BRAF* mutation occurred more frequently in the PTC patients with advanced TNM stage, which is consistent with previous studies [14, 27, 28].

The relationship between *BRAF* mutation and miRNA in PTC had been investigated. However, discrepant results were found from different studies. Chou et al. demonstrated that miRNA-146b was overexpressed in PTC with *BRAF* mutation [29]. Yip et al. also documented that overexpression of miRNA-146b was associated with aggressive behavior in *BRAF* mutant PTC [30]. However, Sheu et al. reported that there was no correlation between *BRAF* mutation and the expression profile of a set of miRNAs (miRNA-221, miRNA-222, miRNA-146b, miRNA-181, and miRNA-21) in PTC [31]. In our study, miRNA-221, miRNA-222, miRNA-146b and miRNA-181 expression levels were significantly higher in PTC patients with *BRAF* mutation. The discrepancy between these studies could be due to different sample origins and the fundamental methodological differences used in the profiling studies. Therefore, it is of interest to further elucidate how these miRNAs and *BRAF* mutation interact with each other in the pathogenesis and progression of PTCs.

miRNA-221 and miRNA-222, which are encoded in tandem on the X chromosome, have been shown to be overexpressed in numerous human tumors including PTC [32–35]. It was reported that p27/kip1 and p57/kip2 were functional targets of miRNA-221 and miRNA-222. Over-expression of miRNA-221 and miRNA-222 in cancer cell lines facilitates cell proliferation via downregulation of the expression of p27/kip1 and/or p57/kip2, both of which are cell cycle inhibitors and tumor suppressors [35]. He et al. have demonstrated the upregulation of miRNA-221 in uninvolved thyroid tissue from individuals with PTC compared to normal thyroid tissue from individuals without thyroid disease [19]. Tetzlaff et al. confirmed that not only miRNA-221 but also miRNA-222 was overexpressed in some cases of multinodular goiter in comparison with PTC [20], suggesting that upregulation of miRNA-221 and miRNA-221 is perhaps early events in thyroid carcinogenesis. Nevertheless, our current study showed that expression of miRNA-221 and miRNA-222 is significantly upregulated in PTC compared to BTN and in patients with advanced TNM stage and lymph node metastasis. Similar to our findings, a report from Taiwan showed that miRNA-221 and miRNA-222 were significantly associated with extrathyroidal invasion of PTC [29]. Taken together, upregulation of miRNA-221 and miRNA-222 may not only be involved in the development of PTC, but also in invasion and metastasis of PTC.

The roles of miR-181 in human carcinogenesis have been explored. miR-181 has been demonstrated to function both as oncogene and tumor suppressor depending on the origin of tissues and cells. Accordingly, miR-181 overexpression has been observed in breast [36] and colon tumors [37]. The downregulation of this miRNA has also been noticed in gliomas [38] and aggressive CLL [39]. In our study, miR-181 expression was significantly higher in PTC versus BTN, suggesting that miR-181 might serve as an oncogene. Moreover, the expression level of miRNA-181 was higher in PTC with larger tumor size. Meng et al. reported that miR-181, by directly targeting on RASSF1A, TIMP3, and NLK, might be involved in hepatocellular cancer stem cells differentiation and invasion [40]. Considering the roles of miR-181 in carcinogenesis, the target genes of miRNA-181 in PTC are required to be identified in the future. 

miRNA-146b over-expression has been shown in the majority of PTC studies [19, 20, 41]. In a series composing of seven pathologically distinct thyroid nodules, Chen et al. demonstrated that miRNA-146b, but not miRNA-221 and miRNA-222, was most consistently overexpressed in PTC [41]. Chou et al. suggested that miR-146b is a novel prognostic factor of PTC, and overexpression of miR-146b significantly increased cell migration and invasiveness [42]. In our study, we showed that miRNA-146b might be more helpful for distinguishing patients with PTC from BTN subjects, while there was no significant difference in miRNA-146b expression in PTC patients with different clinicopathological features. As to the expression levels of miRNA-21, although it was significantly higher in PTC versus BTN, there was substantial expression overlap between the two lesions and there was also no significant difference in miRNA-21 level in PTC patients with any tested clinicopathological features. These miRNAs might act as a tumor-related miRNA, but their roles in the pathogenesis of PTC still need further investigation.

In conclusion, we have confirmed that 5 previously reported miRNAs (miRNA-221, miRNA-222, miRNA-146b, miRNA-181, and miRNA-21) were up-regulated in Chinese patients with PTC. Enhanced expression of these miRNAs occurred more frequently in PTC patients with *BRAF* mutation and some clinicopathological features. Our findings showed a role of selected miRNAs as potential biomarkers in differentiating PTC from BTN and as a prognostic indicator of PTC in Chinese population.

## Figures and Tables

**Table 1:** The clinicopathological features of the study subjects.

Clinicopathological features	Number
Patients with PTC (*n* = 52)	
Age	
<45 years	32
≥45 years	20
Sex	
Male	15
Female	37
Tumor size	
<2 cm	31
≥2 cm	21
TNM staging	
I	38
II	4
III	3
IV	7
Heteromorphic nucleus	6
Multicentricity	20
Tumor location	
Unilateral	45
Bilateral	7
Cervical LN metastasis	29
*BRAF* gene mutation	19
AMES	
Low risk	45
High risk	7
Thyroglobulin level	
Low	3
Normal	35
High	14
Patients with BTN (*n* = 52)	
Age	
<45 years	34
≥45 years	18
Sex	
Male	12
Female	40
Nodule	
Solitary nodule	28
Multinodule	24

TNM: tumor node metastasis; LN: lymph nodes; AMES: age, metastasis to distant sites, extrathyroidal invasion, and tumor size scoring system.

**Table 2:** Differences in miRNA expression in PTC patients with different clinicopathological features.

Clinicopathologic features	miRNA-221		miRNA-222		miRNA-146b		miRNA-181		miRNA-21	
	2^−ΔΔCt^	*P*	2^−ΔΔCt^	*P*	2^−ΔΔCt^	*P*	2^−ΔΔCt^	*P*	2^−ΔΔCt^	*P*
Sex										
Male	9.03 (0.73–269.35)	0.694	7.56 (0.75–58.31)	0.739	129.37 (0.72–552.00)	0.425	5.58 (0.57–12.55)	0.579	10.86 (1.23−38.03)	0.461
Female	8.99 (0.68–212.90)		8.24 (0.04–168.94)		104.69 (1.28–2917.96)		3.15 (0.09–80.72)		7.70 (0.06−168.32)	
Age										
<45 years	8.82 (0.68–212.90)	0.693	7.55 (0.04–168.94)	0.137	103.97 (0.72–2917.96)	0.735	3.38 (0.49–80.72)	0.749	7.07 (1.23−168.32)	0.585
≥45 years	9.29 (0.73–269.35)		13.03 (0.81–58.31)		138.19 (2.36–1123.18)		2.95 (0.09–12.55)		8.32 (0.06−28.83)	
Size										
<2 cm	7.19 (0.73–212.90)	0.004	7.56 (0.75–168.94)	0.244	82.08 (0.72–2917.96)	0.088	2.99 (0.09–80.72)	0.008	6.96 (0.06−168.32)	0.143
≥2 cm	15.60 (0.68–269.35)		10.70 (0.04–58.31)		150.07 (1.28–1123.18)		5.58 (1.21–18.86)		11.24 (1.42−35.14)	
TNM stage										
I/II	8.41 (0.68–212.90)	0.041	7.30 (0.04–168.94)	0.004	92.70 (0.72–2917.96)	0.286	3.12 (0.49–80.72)	0.286	7.26 (1.23−168.32)	0.157
III/IV	21.44 (2.70–269.35)		28.51 (2.73–58.31)		183.96 (5.00–278.56)		4.80 (0.09–12.55)		15.05 (0.06−27.56)	
LN metastasis										
No	6.68 (0.73–212.90)	0.033	4.54 (0.75–22.92)	0.014	82.15 (0.72–2917.96)	0.250	2.36 (0.57–80.72)	0.103	7.17 (1.23−168.32)	0.423
Yes	14.43 (0.68–269.35)		11.82 (0.04–168.94)		129.37 (1.28–1123.18)		4.35 (0.09–18.86)		9.82 (0.06–28.83)	
*BRAF* mutation										
No	6.36 (0.68–269.35)	0.001	4.73 (0.04–168.94)	0.019	55.11 (0.72–2917.96)	0.003	2.46 (0.09–80.72)	0.005	6.81 (0.06–168.32)	0.104
Yes	15.29 (2.70–62.02)		11.82 (2.73–43.93)		182.19 (10.70–1123.18)		5.58 (2.08–18.86)		10.86 (1.44–28.83)	
Heteromorphic nucleus										
No	9.16 (0.68–269.35)	0.819	10.39 (0.04–168.94)	0.302	118.72 (0.72–2917.96)	0.240	3.38 (0.09–80.72)	0.797	8.32 (0.06–168.32)	0.548
Yes	8.82 (0.86–18.00)		6.13 (1.94–8.24)		49.69 (6.77–144.74)		3.12 (0.49-9.49)		5.46 (1.44-39.03)	
Multicentricity										
No	8.33 (0.73–212.90)	0.807	7.94 (0.75–168.94)	0.940	92.67 (0.72–2917.96)	0.807	3.26 (0.49–0.72)	0.866	9.00 (1.23–168.32)	0.275
Yes	10.01 (0.68–269.35)		7.63 (0.04–58.31)		118.72 (1.28–313.67)		3.23 (0.09–18.86)		7.07 (0.06–35.14)	
AMES										
Low	8.99 (0.68–212.90)	0.341	7.65 (0.04–168.94)	0.203	8	0.862	3.15 (0.09–80.72)	0.266	7.60 (0.06–168.32)	0.583
High	22.82 (0.73–269.35)		37.91 (1.67–58.31)		150.07 (2.36–278.56)		5.59 (0.57–12.55)		8.75 (1.30–27.40)	
Thyroglobulin level										
Low	7.67 (0.68–21.50)	0.225	12.23 (0.82–13.37)	0.626	205.95 (1.28–208.88)	0.427	2.46 (1.21–4.35)	0.993	17.55 (1.42–27.56)	0.407
Normal	8.17 (0.73–212.90)		7.65 (0.04–168.94)		103.26 (0.72–2917.96)		3.15 (0.09–80.72)		7.17 (0.06–168.32)	
High	13.01 (1.14–269.35)		10.82 (0.81–58.31)		136.74 (6.75–1123.18)		3.44 (0.79–12.55)		8.89 (3.91–28.83)	

The data are shown as median (range); *P* value was determined by the Mann-Whitney *U *test.

TNM: tumor node metastasis; LN: lymph nodes; AMES: age, metastasis to distant sites, extrathyroidal invasion, and tumor size scoring system.

## References

[1] Sipos JA, Mazzaferri EL (2010). Thyroid cancer epidemiology and prognostic variables. *Clinical Oncology*.

[2] Lukas J, Drabek J, Lukas D (2012). The epidemiology of thyroid cancer in the Czech Republic in comparison with other countries. *Biomedical papers of the Medical Faculty of the University Palacký, Olomouc, Czechoslovakia*.

[3] Liu S, Semenciw R, Ugnat AM, Mao Y (2001). Increasing thyroid cancer incidence in Canada, 1970–1996: time trends and age-period-cohort effects. *British Journal of Cancer*.

[4] Burgess JR (2002). Temporal trends for thyroid carcinoma in Australia: an increasing incidence of papillary thyroid carcinoma (1982–1997). *Thyroid*.

[5] Burgess JR, Tucker P (2006). Incidence trends for papillary thyroid carcinoma and their correlation with thyroid surgery and thyroid fine-needle aspirate cytology. *Thyroid*.

[6] Lubina A, Cohen O, Barchana M (2006). Time trends of incidence rates of thyroid cancer in Israel: what might explain the sharp increase. *Thyroid*.

[7] Colonna M, Grosclaude P, Remontet L (2002). Incidence of thyroid cancer in adults recorded by French cancer registries (1978–1997). *European Journal of Cancer*.

[8] Leenhardt L, Grosclaude P, Chérié-Challine L (2004). Increased incidence of thyroid carcinoma in france: a true epidemic or thyroid nodule management effects? Report from the french thyroid cancer committee. *Thyroid*.

[9] Davies L, Welch HG Increasing incidence of thyroid cancer in the United States, 1973–2002. *JAMA*.

[10] Hundahl SA, Fleming ID, Fremgen AM (1998). A National Cancer Data Base report on 53,856 cases of thyroid carcinoma treated in the U.S. 1985–1995. *Cancer*.

[11] Xing M (2005). *BRAF* mutation in thyroid cancer. *Endocrine-Related Cancer*.

[12] Xing M (2007). *BRAF* mutation in papillary thyroid cancer: pathogenic role, molecular bases, and clinical implications. *Endocrine Reviews*.

[13] Li X, Abdel-Mageed AB, Kandil E (2012). *BRAF* mutation in papillary thyroid carcinoma. *International Journal of Clinical and Experimental Medicine*.

[14] Xing M, Westra WH, Tufano RP (2005). *BRAF* mutation predicts a poorer clinical prognosis for papillary thyroid cancer. *The Journal of Clinical Endocrinology & Metabolism*.

[15] Lupi C, Giannini R, Ugolini C (2007). Extensive clinical experience: association of *BRAF* V600E mutation with poor clinicopathological outcomes in 500 consecutive cases of papillary thyroid carcinoma. *The Journal of Clinical Endocrinology & Metabolism*.

[16] Kim SJ, Lee KE, Myong JP (2012). BRAF^V600E^ mutation is associated with tumor aggressiveness in papillary thyroid cancer. *World Journal of Surgery*.

[17] Esquela-Kerscher A, Slack FJ (2006). Oncomirs-microRNAs with a role in cancer. *Nature Reviews Cancer*.

[18] Pallante P, Visone R, Ferracin M (2006). MicroRNA deregulation in human thyroid papillary carcinomas. *Endocrine-Related Cancer*.

[19] He H, Jazdzewski K, Li W (2005). The role of microRNA genes in papillary thyroid carcinoma. *Proceedings of the National Academy of Sciences of the United States of America*.

[20] Tetzlaff MT, Liu A, Xu X (2007). Differential expression of miRNAs in papillary thyroid carcinoma compared to multinodular goiter using formalin fixed paraffin embedded tissues. *Endocrine Pathology*.

[21] Weber F, Teresi RE, Broelsch CE, Frilling A, Eng C (2006). A limited set of human MicroRNA is deregulated in follicular thyroid carcinoma. *The Journal of Clinical Endocrinology & Metabolism*.

[22] Lodewijk L, Prins AM, Kist JW (2012). The value of miRNA in diagnosing thyroid cancer: a systematic review. *Cancer Biomark*.

[23] Xing M, Tufano RP, Tufaro AP (2004). Detection of *BRAF* mutation on fine needle aspiration biopsy specimens: a new diagnostic tool for papillary thyroid cancer. *The Journal of Clinical Endocrinology & Metabolism*.

[24] Livak KJ, Schmittgen TD (2001). Analysis of relative gene expression data using real-time quantitative PCR and the 2-ΔΔCT method. *Methods*.

[25] Bakhos R, Selvaggi SM, DeJong S (2013). Fine needle aspiration of the thyroid: rate and causes of cytopathologic discordance. *Diagnostic Cytopathology*.

[26] Sung JY, Na DG, Kim KS (2012). Diagnostic accuracy of fine-needle aspiration versus core-needle biopsy for the diagnosis of thyroid malignancy in a clinical cohort. *European Radiology*.

[27] Nikiforova MN, Kimura ET, Gandhi M (2003). *BRAF* mutations in thyroid tumors are restricted to papillary carcinomas and anaplastic or poorly differentiated carcinomas arising from papillary carcinomas. *The Journal of Clinical Endocrinology & Metabolism*.

[28] Namba H, Nakashima M, Hayashi T (2003). Clinical implication of hot spot *BRAF* mutation V599E, in papillary thyroid cancers. *The Journal of Clinical Endocrinology & Metabolism*.

[29] Chou CK, Chen RF, Chou FF (2010). MiR-146b is highly expressed in adult papillary thyroid carcinomas with high risk features including extrathyroidal invasion and the *BRAF* V600E mutation. *Thyroid*.

[30] Yip L, Kelly L, Shuai Y (2011). MicroRNA Signature Distinguishes the Degree of Aggressiveness of Papillary Thyroid Carcinoma. *Annals of Surgical Oncology*.

[31] Sheu SY, Grabellus F, Schwertheim S, Handke S, Worm K, Schmid KW (2009). Lack of correlation between *BRAF* V600E mutational status and the expression profile of a distinct set of miRNAs in papillary thyroid carcinoma. *Hormone and Metabolic Research*.

[32] Miller TE, Ghoshal K, Ramaswamy B (2008). MicroRNA-221/222 confers tamoxifen resistance in breast cancer by targeting p27Kip1. *The Journal of Biological Chemistry*.

[33] Yoon SO, Chun SM, Han EH (2011). Deregulated expression of microRNA-221 with the potential for prognostic biomarkers in surgically resected hepatocellular carcinoma. *Human Pathology*.

[34] Lee EJ, Gusev Y, Jiang J (2007). Expression profiling identifies microRNA signature in pancreatic cancer. *International Journal of Cancer*.

[35] Visone R, Russo L, Pallante P (2007). MicroRNAs (miR)-221 and miR-222, both overexpressed in human thyroid papillary carcinomas, regulate p27Kip1 protein levels and cell cycle. *Endocrine-Related Cancer*.

[36] Yan LX, Huang XF, Shao Q (2008). MicroRNA miR-21 overexpression in human breast cancer is associated with advanced clinical stage, lymph node metastasis and patient poor prognosis. *RNA*.

[37] Nakajima G, Hayashi K, Xi Y (2006). Non-coding microRNAs hsa-let-7g and hsa-miR-181b are associated with chemoresponse to S-1 in colon cancer. *Cancer Genomics and Proteomics*.

[38] Shi L, Cheng Z, Zhang J (2008). hsa-mir-181a and hsa-mir-181b function as tumor suppressors in human glioma cells. *Brain Research*.

[39] Calin GA, Pekarsky Y, Croce CM (2007). The role of microRNA and other non-coding RNA in the pathogenesis of chronic lymphocytic leukemia. *Best Practice and Research in Clinical Haematology*.

[40] Meng F, Glaser SS, Francis H (2012). Functional Analysis of microRNAs in Human Hepatocellular Cancer Stem Cells. *Journal of Cellular and Molecular Medicine*.

[41] Chen YT, Kitabayashi N, Zhou XK (2008). MicroRNA analysis as a potential diagnostic tool for papillary thyroid carcinoma. *Modern Pathology*.

[42] Chou CK, Yang KD, Chou FF (2013). Prognostic implications of miR-146b expression and its functional role in papillary thyroid carcinoma. *The Journal of Clinical Endocrinology & Metabolism*.

